# The performance of the node reporting and data system 1.0 (Node-RADS) and DWI–MRI in staging patients with cervical carcinoma according to the new FIGO classification (2018)

**DOI:** 10.1007/s11547-024-01824-9

**Published:** 2024-05-10

**Authors:** Roberta Valerieva Ninkova, Alessandro Calabrese, Federica Curti, Sandrine Riccardi, Marco Gennarini, Valentina Miceli, Angelica Cupertino, Violante Di Donato, Angelina Pernazza, Stefania Maria Rizzo, Valeria Panebianco, Carlo Catalano, Lucia Manganaro

**Affiliations:** 1 Department of Radiological, Oncological and Pathological Sciences, Sapienza University of Rome, Policlinico Umberto I, Viale del Policlinico 155, 00161 Rome, Italy; 2 Department of Maternal and Child Health and Urological Sciences, Oncological and Pathological Sciences, Sapienza, University of Rome, Policlinico Umberto I, Viale del Policlinico 155, 00161 Rome, Italy; 3 Faculty of Biomedical Sciences, University of Italian Switzerland (USI), Via Buffi 13, 6900 Lugano, Switzerland; 4 Service of Radiology, Imaging Institute of Southern Switzerland, Clinica Di Radiologia EOC, 6900 Lugano, Switzerland

**Keywords:** Magnetic resonance imaging, Lymph node, Uterine cervical cancer, Apparent diffusion coefficient

## Abstract

**Purpose:**

To evaluate the diagnostic accuracy of the Node-RADS score and the utility of apparent diffusion coefficient (ADC) values in predicting metastatic lymph nodes (LNs) involvement in cervical cancer (CC) patients using magnetic resonance imaging (MRI). The applicability of the Node RADS score across three readers with different years of experience in pelvic imaging was also assessed.

**Material and methods:**

Among 140 patients, 68 underwent staging MRI, neoadjuvant chemotherapy and radical surgery, forming the study cohort. Node-RADS scores of the main pelvic stations were retrospectively determined to assess LN metastatic likelihood and compared with the histological findings. Mean ADC, relative ADC (rADC), and correct ADC (cADC) values of LNs classified as Node-RADS ≥ 3 were measured and compared with histological reports, considered as gold standard.

**Results:**

Sensitivity, specificity, positive and negative predictive values (PPVs and NPVs), and accuracy were calculated for different Node-RADS thresholds. Node RADS ≥ 3 showed a sensitivity of 92.8% and specificity of 72.5%. Node RADS ≥ 4 yielded a sensitivity of 71.4% and specificity of 100%, while Node RADS 5 yielded 42.9% and 100%, respectively. The diagnostic performance of mean ADC, cADC and rADC values from 78 LNs with Node-RADS score ≥ 3 was assessed, with ADC demonstrating the highest area under the curve (AUC 0.820), compared to cADC and rADC values.

**Conclusion:**

The Node-RADS score provides a standardized LNs assessment, enhancing diagnostic accuracy in CC patients. Its ease of use and high inter-observer concordance support its clinical utility. ADC measurement of LNs shows promise as an additional tool for optimizing patient diagnostic evaluation.

## Introduction

Cervical cancer (CC) is the fourth leading cause of mortality among women worldwide, with an estimated annual incidence of 470,000 new cases. In 2017, the global incidence of CC reached approximately 0.6 million cases, resulting in 8.1 million disability-adjusted life-years (DALYs) lost and 0.26 million deaths [1].

In 2018, the International Federation of Gynecology and Obstetrics (FIGO) revised the staging system for CC, highlighting the important role of imaging for loco-regional staging and lymph node (LN) involvement, introducing stage IIIC, further divided into IIIC1 (pelvic LNs metastasis) and IIIC2 (para-aortic LNs metastases) [2–4]. Consequently, an accurate assessment of metastatic LNs is critical to determine the optimal treatment and predicting prognosis.

Elsholtz et al. proposed the node reporting and data system 1.0 (Node-RADS) to standardize the radiological assessment of LN involvement using computed tomography (CT) and magnetic resonance (MR) imaging. Node-RADS is based on both size and configuration criteria to assign a 5-point assessment category and is applicable to tumors in any anatomical site [5]. Although Node-RADS has shown promising results in prostate and bladder cancer, its role in CC has not been explored [6, 7].

Currently, MRI is preferred for assessing loco-regional staging of CC due to its superior soft tissue contrast and multiplanar imaging capability. Additionally, MRI allows quantitative evaluation using diffusion-weighted imaging (DWI) and Apparent Diffusion Coefficient (ADC) mapping [8], which have proven useful in identifying metastatic LNs [9].

For this purpose, we conducted a retrospective analysis of preoperative MRI data with the following aims: first, to evaluate the overall diagnostic performance of the Node-RADS score in predicting the likelihood of LN involvement in CC patients by validating radiological data with post-surgical anatomopathological findings obtained through lymphadenectomy. Secondly, to explore the utility of ADC measurement in characterizing pelvic metastatic LNs in CC patients. Lastly, to assess the applicability and feasibility of the scoring system across three different readers with different years of experience in female pelvic imaging.

## Material and methods

### Study protocol

This study is a retrospective analysis conducted at a single center. Written informed consent was obtained for data collection from each patient included in the study. The research methods and protocols adhered to the ethical standards outlined by our institution and research committee, following the principles of the 2013 Declaration of Helsinki and the latest amendments.

### Patient population

From January 2015 to October 2023, a total of 140 patients with CC received treatment at our institution. Clinical data on age and human papillomavirus (HPV) status were systematically collected.

Inclusion criteria for patient selection were as follows: (a) confirmation of histologic diagnosis indicating squamous cell carcinoma, adenocarcinoma, or neuroendocrine carcinoma of the cervix; (b) pre-operative staging MRI with DWI sequences; (c) radical surgery followed by postoperative histologic LN analysis after neoadjuvant chemotherapy (NACT).

Patients with concurrent or previous malignancies, those lacking preoperative MRI assessments, patient who did not undergo surgery, and those with incomplete histological reports were excluded from the study.

### MRI technique

MRI examinations were performed on a 3 Tesla (3T) magnet (GE Discovery 750; Siemens, Siemens VIDA) using a 32-channels phased-array body coil or a 16-channel phased-array coil positioned over the lower abdomen.

The study protocol required patients to void their bladders one hour before the radiological examination to ensure optimal bladder distention. In addition, fasting before the examination was recommended. Prior to the start of the MRI examination, intravenous scopolamine-N-butylbromide (Buscopan; Boehringer Ingelheim, Ingelheim, Germany) was administered to all patients, unless contraindicated, to reduce bowel peristalsis artifacts.

The standard MRI protocol, focusing on the lower abdominal region from the pubic symphysis to the iliac crests, included the following sequences: T2-weighted fast spin-echo (FSE) with thin sections (3 mm) and a field of view (FOV) of 20–24 mm for optimal anatomical resolution, acquired in sagittal, axial, para-axial, and para-coronal planes; T1-weighted FSE and fat saturated T1-weighted in the axial plane; axial and para-axial diffusion-weighted images (DWI) with diffusion-sensitizing gradient with a *b*-value of 0–1000 s/mm^2^ to generate the apparent diffusion coefficient (ADC) maps on a post-processing workstation (AW Volume-Share 7, GE Healthcare, Milwaukee, WI, USA); dynamic T1-weighted 3D gradient-echo sequences with fat saturation in the axial plane during contrast uptake, followed by delayed post-contrast T1-weighted 3D gradient echo sequences with fat saturation in the axial plane (Table 1).

**Table 1:** MR protocol

	Magnet	TR/TE (ms)	FOV (mm)	NEX	Matrix size	Slice thickness (mm)	Intersection gap (mm)	B values (s/mm^2^)	FA (°)	Temporal resolution (s)
Sagittal FSE T2WI	GE	5733/120	240 × 240	6	256 × 224	3	0.5	–	110	–
	VIDA	9460/110	220 × 220	2	352 × 264	3	0.3	–	160	–
Para-axial, Para-coronal FSE T2WI	GE	4500/120	240 × 240	6	256 × 224	3	0.5	–	110	–
	VIDA	8900/134	220 × 200	2	352 × 240	3	0.3	–	160	–
Axial FSE T2WI	GE	5495/120	240 × 240	6	320 × 320	3	0.5	–	110	–
	VIDA	9460/110	220 × 220	2	352 × 264	3	0.3	–	160	–
Axial FSE T1WI	GE	500/42	240 × 240	4	320 × 224	3	0.5	–	110	–
	VIDA	480/20	220 × 220	2	352 × 264	3	0.3	–	150	–
Axial T1WI LAVA	GE	500/42	240 × 240	4	320 × 224	3	0.5	–	110	–
Axial T1WI DIXON	VIDA	5.6/2.46	340 × 255	1	384 × 202	0.9	0	–	9	–
Para-axial DWI	GE	3500/60	240 × 240	2–4–6	100 × 100	3	0.5	50–500–1000	–	–
	VIDA	6500/93	220/220	2–4–6	96 × 96	3	0.3	0–500–1000	–	–
Axial DWI	GE	5400/60	240 × 240	2–4–6	100 × 100	3	0.5	50–500–1000	–	–
	VIDA	8300/93	280	1–2–4	10 × 110	3	0.8	0–500–1000	–	–
3D-DCE T1WI	GE	2.7/1.2	280 × 280	1	160 × 140	3	/	/	15	10
	VIDA	4.46/1.48	220 × 220	1	144 × 160	3	/	/	20	6

TR, repetition time; TE, echo time; FOV, field of view; NEX, number of excitations; FA, flip angle; WI, weighted imaging; FSE, fast spin-echo; FS, fat saturation; DCE, dynamic contrast enhanced

Gadolinium-based contrast agent (gadoteric acid, Dotarem®) was administered intravenously at a dose of 0.2 mL/kg through peripheral venous access (22-gauge) using a power injector at a rate of 2 mL/s, followed by a saline flush of 20 mL. Post-contrast images were acquired sequentially at 6–10-s intervals, starting 10 s before bolus injection, with a total acquisition time of 320 s.

### Images analysis

**Fig. 1:**
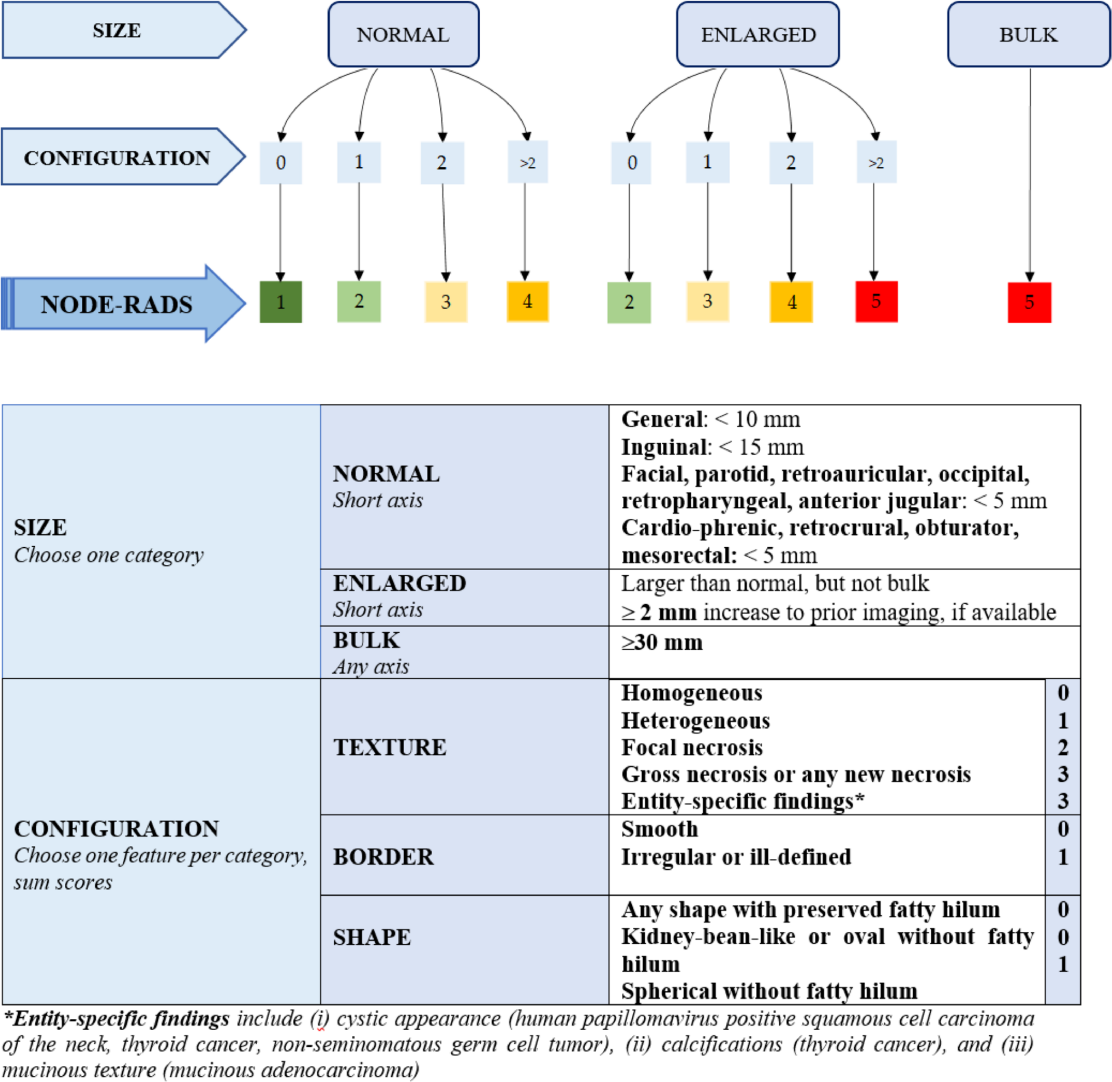
Node-RADS flowchart with a description of size and configuration criteria for lymph node assessment

**Fig. 2:**
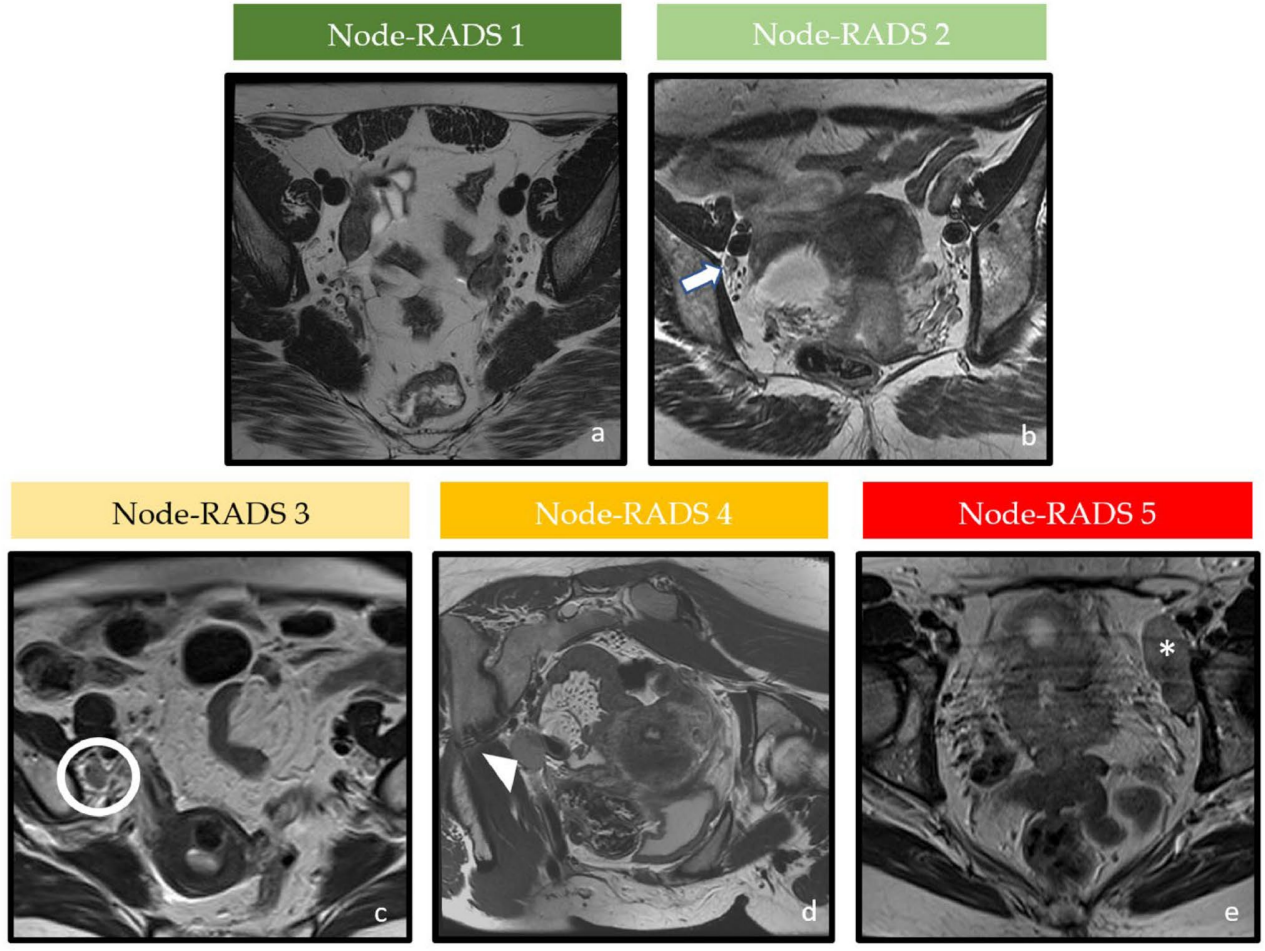
Axial T2-weighted images of pelvic lymph nodes with different Node-RADS score according to suspected malignancy. **a** Node-RADS 1: left external iliac LN with normal size and configuration. **b** Node-RADS 2: left external iliac LN (arrow), showing a short axis of 5 mm and homogeneous signal intensity. **c** Node-RADS 3: the left external iliac LN (circle), showing a short axis of 8 mm, heterogeneous signal intensity with oval shape without fatty hilum. **d** Node-RADS 4: left external iliac LN (arrow), showing a short axis of 13 mm with heterogeneous signal intensity and spherical shape without fatty hilum. **e** Node-RADS 5: right obturator bulky LN (asterisk) with long axis > 30 mm, corresponding to a high risk of malignancy.

Three readers, including two radiologists with different experience in pelvic MRI (1 and 4 years, respectively), and a senior radiologist with extensive experience (25 years) in pelvic MRI, independently reviewed the images and assigned a Node-RADSscore to each pelvic LN bilaterally (common iliac, internal iliac, external iliac and obturator), blinded to postoperative histopathological results. The image analysis was performed according to Node-RADS recommendations (Figs. 1, 2).

Node-RADS assessment incorporates categories based on “size” and “configuration” criteria, each with associated subcategories (see Fig. 1). The Node-RADS score classifies LNs into normal, enlarged, and bulky based on the “size”. “configuration” criteria involve morphological examination of LNs, such as texture, border and shape subcategories (see Fig. 1). The “configuration” score results from the sum of the values assigned to each subcategory.

### ADC-based evaluation

The LNs assessed on axial T2-weighted images were visualized on axial pelvic DWI and the corresponding ADC map. ADC values were manually determined by placing three specific 3–6 mm circular regions of interest (ROIs) on each LN categorized as indeterminate to very high malignancy risk (Node-RADS 3–5), and both within the tumor and on the gluteus muscle. Specifically, three ADC values were calculated: the mean ADC (ADC), representing the ADC value of the suspect LN; the corrected ADC (cADC), derived from the ratio of the LN ADC to the ADC of the right gluteus maximum muscle (ADC/gluteus ADC); and the ADC ratio (rADC), calculated by subtracting the mean ADC value of the primary tumor from that of the LN (ADC-main tumor ADC).

### Pathological analysis

The following characteristics were collected for each patient: surgical margins infiltration, surgical parametric infiltration, postoperative FIGO stage, grade, histological type and pN status.

In clinical practice there are no established guidelines on how to process and evaluate resected CC specimens after NACT, although a comprehensive mapping approach to gross and histologic processing after NACT has been proposed for other kinds of tumor.

Pathologists identified the area where the original pre-treatment tumor was considered to be located, also known as “tumor bed”, in both in the resection specimen and LNs. To this purpose, we compared the pretherapy and preoperative radiologic images with the gross features. After identifying the tumor bed in the cervix specimen, it was sectioned and totally submitted in order to demonstrate the presence of residual viable tumor and its relationship to the surrounding structures relevant for staging and the surgical resection margin. LNs were sectioned and totally submitted to evaluate the presence of metastases or related changes due to therapy.

Regarding LNs, we identified the same reactive alterations present in the tumor bed area of the resection specimen, such us sclerosis, hyalinized connective tissue, histiocytes, xanthogranulomatous or granulomatous reaction and cholesterol clefts.

Then, the following were reported: LN stations with treatment-related changes without viable tumor, LN stations involved by tumor with treatment related changes (recording largest tumor focus and extracapsular extension), LN stations without treatment-related changes without viable tumor.

**Fig. 3:**
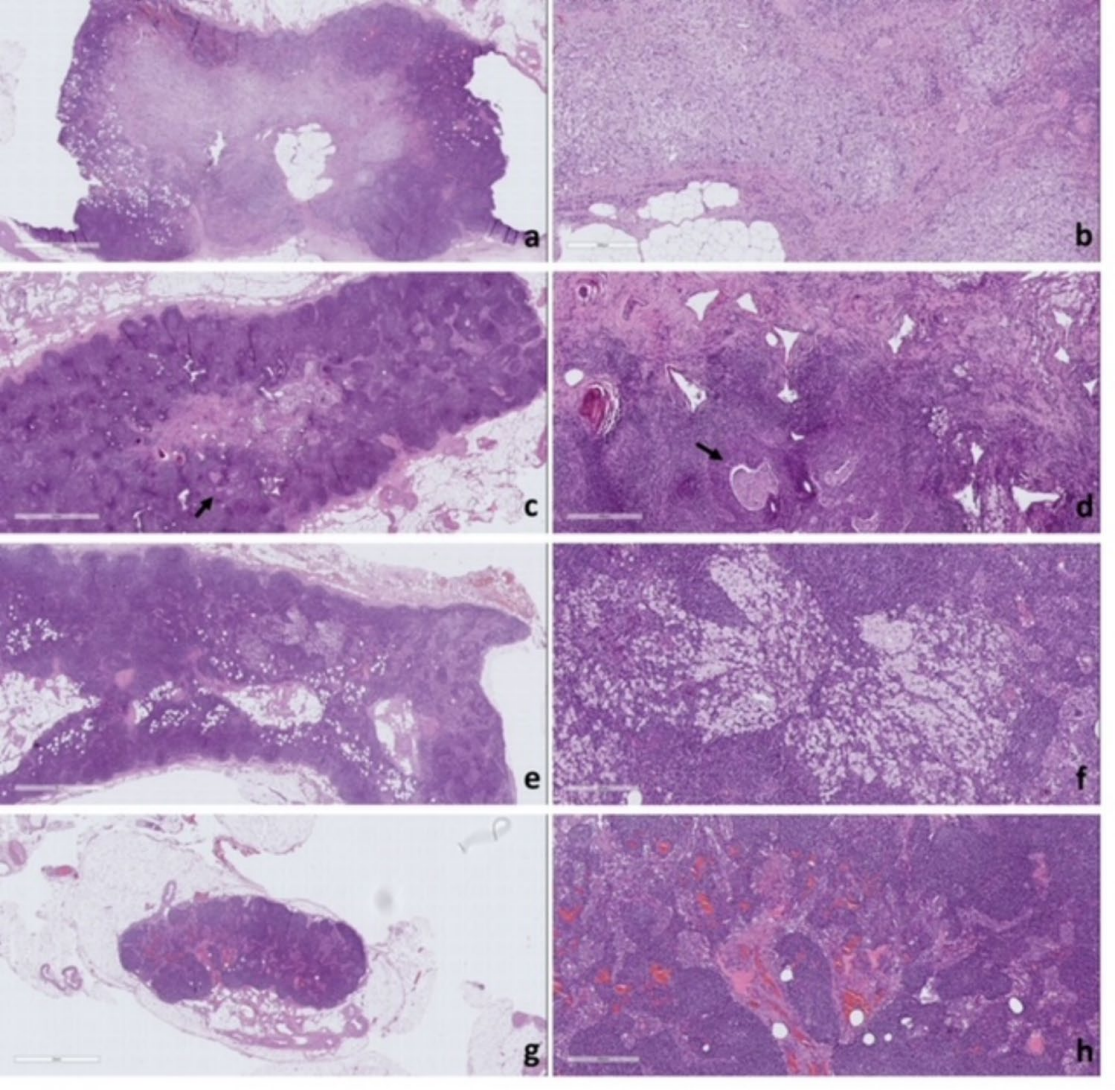
Lymph node with treatment-related changes without viable tumor: tumor bed area was entirely composed of accumulation of foamy histiocytes associated with sclerosis (**a** low magnification; **b** high magnification). Lymph node involved by tumor (arrow) with treatment related changes: this lymph node contains a focus of metastatic squamous cell carcinoma. It is difficult to be certain what is inflammation in the stroma because of the background lymphocytes in the lymph node. However, the presence of pools of foamy histiocytes with stromal fibrosis confirmed the presence of regressive alteration (**c** low magnification; **d** high magnification). Lymph node with treatment-related changes without viable tumor: tumor bed area was entirely composed of accumulation of foamy histiocytes without sclerosis (**e** low magnification; **f** high magnification). Lymph node stations without treatment-related changes without viable tumor: note the normal parenchyma with sinus histiocytosis (**g** low magnification; **h** high magnification).

Histological data were compared with the radiological findings to assess the validity of the assigned Node-RADS score (Fig. 3).

### Statistical analysis

Spearman’s rank-order correlation test was performed to determine the correlation between the highest Node-RADS score value assigned to each patient by the senior reader and pN status, and the other clinical and radiological qualitative features (histologic type, grade, positive margins at surgery, parametric infiltration at surgery, pre- and postoperative FIGO stage, LN size < 10 mm vs LN size ≥ 10 mm).

The Shapiro–Wilk test was performed to determine whether quantitative features (age and tumor size) followed a normal distribution. A one-way analysis of variance (ANOVA) test was performed to determine whether there were any statistically significant differences of the quantitative features for the five Node-RADS scores.

A receiver operating characteristic (ROC) curve was performed to calculate the area under the ROC curve (AUC) to evaluate the diagnostic performance of the Node-RADS score in predicting pN status.

Sensitivity, specificity, positive predictive value (PPV), negative predictive value (NPV), and accuracy for the 4 possible cut offs (≥ 2, ≥ 3, ≥ 4, 5) were obtained.

Cohen’s κ was used to determine inter-reader agreement between Node-RADS scores assigned by the senior reader vs. scores assigned by junior reader 1, and the scores assigned by the senior reader vs junior reader 2.

The Shapiro–Wilk test was performed to determine whether ADC, cADC and rADC values followed a normal distribution. Since not all the analyzed variables followed a normal distribution, a Kruskal–Wallis *H* test was performed to determine whether there were any statistically significant differences of the ADC, cADC and rADC values for Node-RADS 3, 4 and 5.

ROC curves were calculated to evaluate the diagnostic performance in predicting pN status of ADC, cADC and rADC of all LNs with Node-RADS score value ≥ 3. Next, a ROC curve was performed to evaluate the diagnostic performance of ADC, cADC and rADC of all LNs with Node-RADS score value 3 in predicting pN status. The cutoff values for ADC, cADC and rADC were assigned using Youden’s index. Sensitivity, specificity, PPV, NPV and accuracy for ADC, cADC and rADC were obtained for the Node-RADS ≥ 3 group and for Node-RADS 3.

Statistical significance was set at *p* < 0.05. All data analyses were processed using Statistical Package for the Social Sciences software (SPSS Statistic version 25.0, SPSS, Chicago, IL, USA).

## Results

**Fig. 4:**
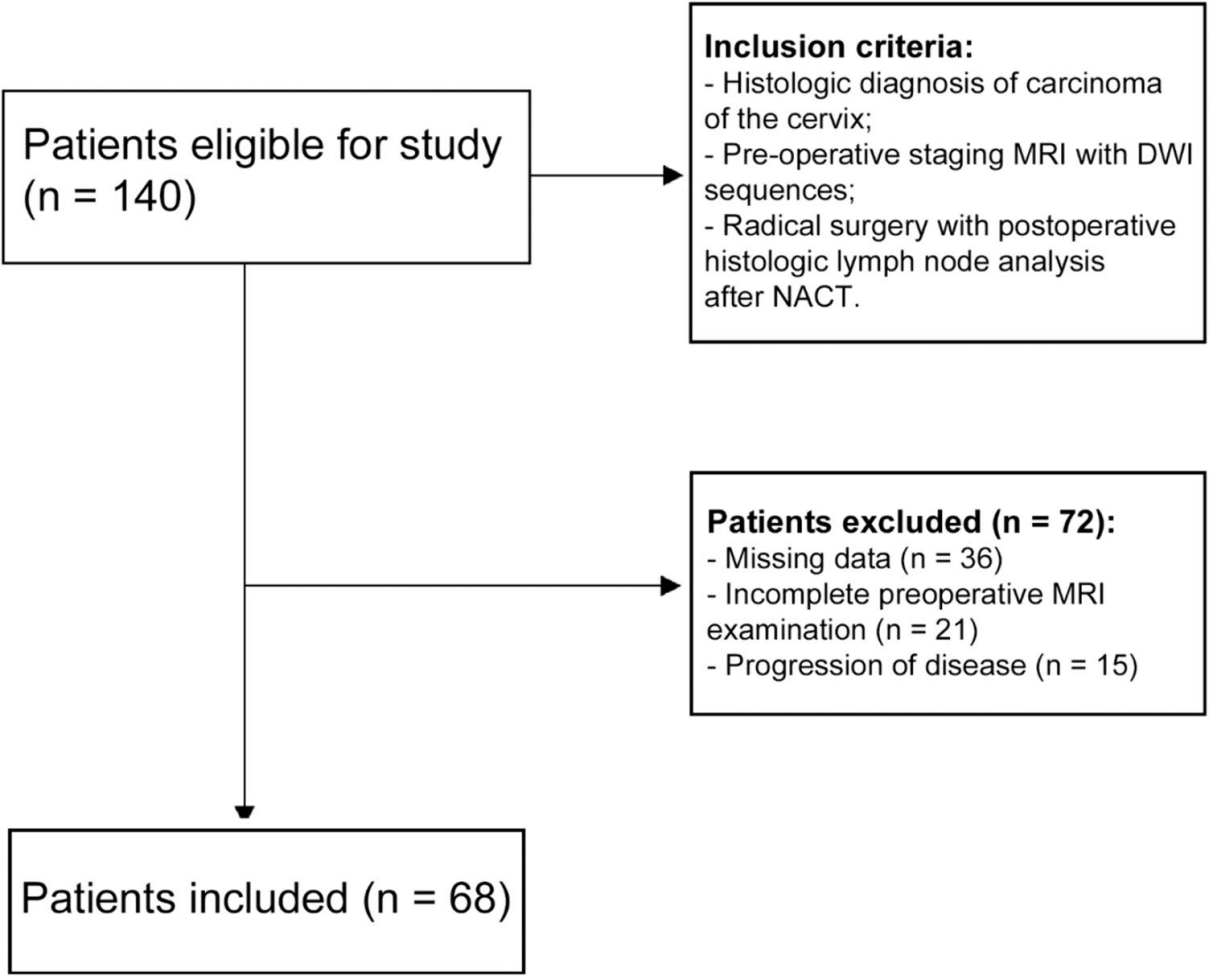
Flowchart of patient inclusion. (MRI = magnetic resonance imaging; DWI: diffusion-weighted imaging; NACT: neoadjuvant chemotherapy)

From a database of 140 patients who underwent MRI examination between January 2015 and October 2023, a total of 68 patients with histologically proven locally advanced CC met the inclusion criteria and their MRI images were retrospectively reviewed. Seventy-two patients were excluded from our study due to missing data, incomplete preoperative MRI examination, or with disease progression (Fig. 4).

The mean age was 56 years (range 31–84 years). Of the included patients, 40/68 (58.8%) had no LN infiltration on definitive histologic examination (pN = 0), while 28/68 (42.2%) had at least one positive LN (pN ≥ 1) in one of the eight pelvic LN stations evaluated.

The senior reader assigned Node-RADS scores of 1, 2, 3, 4 and 5 to 18/68 (26.5%), 13/68 (19.1%), 17/68 (25.0%), 8/68 (11.8%) and 12/68 (17.6%) patients, respectively.

The frequencies of qualitative clinical and radiological features are summarized in Table 1. A positive and significant correlation was found between Node-RADS score and pN status (rs = 0.771, *p* < 0.001), positive resection margins (rs = 0.289, p = 0.021), pre-operative FIGO stage at MRI (rs = 0.353, *p* = 0.003), postoperative FIGO stage at MRI (rs = 0.563, *p* < 0.001) and LN size (rs = 0.489, *p* < 0.001).

**Fig. 5:**
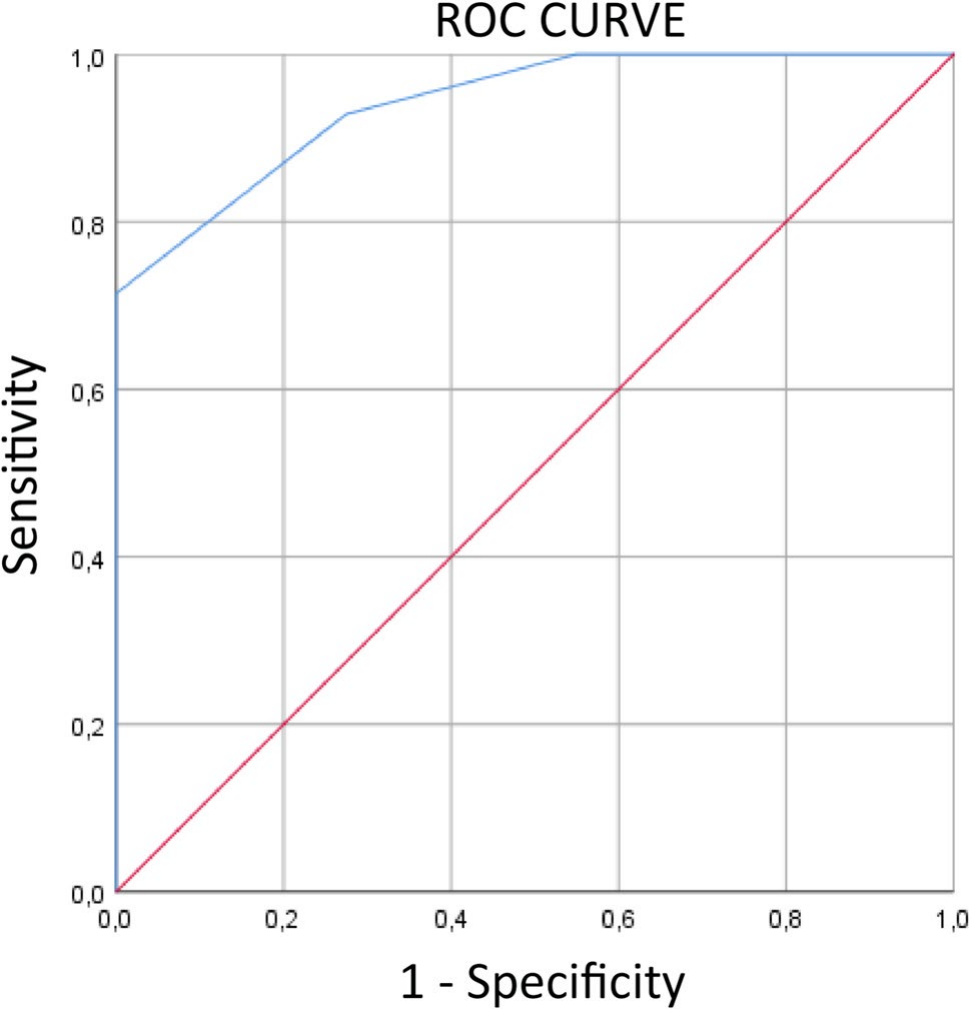
Receiver operating characteristic (ROC) curve for the prediction of pN status using the Node-RADS score. Area under the curve (AUC) = 0.941

The ANOVA test showed that only tumor size was significantly different among the five Node-RADS score groups (*F* = 2.760, *p* = 0.036) (Table 2). Using the Node-RADS scores, the prediction of pN status corresponded to an AUC of 0.941 (Fig. 5). Node-RADS score performance in predicting pN status in cervical cancer is reported in Table 3.

**Table 2:** Clinical, pathological, MRI qualitative and quantitative characteristics of the study population

			NODE-RADS						*p*-value
			1	2	3	4	5	Total	
HPV Status	Negative	*N*	12	6	10	2	6	36	0.168
		%	17.6%	8.8%	14.7%	2.9%	8.8%	52.9%	
	Positive	*N*	6	7	7	6	6	32	
		%	8.8%	10.3%	10.3%	8.8%	8.8%	47.1%	
Lymph node size	< 10 mm	*N*	17	11	4	0	0	32	** < 0.001**
		%	25.0%	16.2%	5.9%	0.0%	0.0%	47.1%	
	≥ 10 mm	*N*	1	2	13	8	12	36	
		%	1.5%	2.9%	19.1%	11.8%	17.6%	52.9%	
Pre-operative FIGO Stage	IB1	N	3	0	0	0	0	3	**0.003**
		%	4.4%	0.0%	0.0%	0.0%	0.0%	4.4%	
	IB2	*N*	0	1	0	0	0	1	
		%	0.0%	1.5%	0.0%	0.0%	0.0%	1.5%	
	IB3	*N*	1	0	0	0	0	1	
		%	1.5%	0.0%	0.0%	0.0%	0.0%	1.5%	
	IIA1	*N*	2	0	1	0	0	3	
		%	2.9%	0.0%	1.5%	0.0%	0.0%	4.4%	
	IIB	*N*	11	5	5	0	0	21	
		%	16.2%	7.4%	7.4%	0.0%	0.0%	30.9%	
	IIIB	*N*	0	0	1	0	0	1	
		%	0.0%	0.0%	1.5%	0.0%	0.0%	1.5%	
	IIIC1	*N*	0	6	8	7	10	31	
		%	0.0%	8.8%	11.8%	10.3%	14.7%	45.6%	
	IIIC2	*N*	0	0	2	1	0	3	
		%	0.0%	0.0%	2.9%	1.5%	0.0%	4.4%	
	IVA	*N*	1	0	0	0	2	3	
		%	1.5%	0.0%	0.0%	0.0%	2.9%	4.4%	
	IVB	*N*	0	1	0	0	0	1	
		%	0.0%	1.5%	0.0%	0.0%	0.0%	1.5%	
Postoperative FIGO Stage	IA1	*N*	2	1	1	0	0	4	** < 0.001**
		%	4.1%	2.0%	2.0%	0.0%	0.0%	8.2%	
	IA2	*N*	1	2	2	0	0	5	
		%	2.0%	4.1%	4.1%	0.0%	0.0%	10.2%	
	IB1	*N*	5	4	2	2	2	15	
		%	10.2%	8.2%	4.1%	4.1%	4.1%	30.6%	
	IB2	*N*	2	0	4	0	0	6	
		%	4.1%	0.0%	8.2%	0.0%	0.0%	12.2%	
	IIA1	*N*	0	2	2	1	0	5	
		%	0.0%	4.1%	4.1%	2.0%	0.0%	10.2%	
	IIA2	*N*	1	0	3	0	1	5	
		%	2.0%	0.0%	6.1%	0.0%	2.0%	10.2%	
	IIB	*N*	0	0	2	0	0	2	
		%	0.0%	0.0%	4.1%	0.0%	0.0%	4.1%	
	IIIB	*N*	0	1	1	2	2	6	
		%	0.0%	2.0%	2.0%	4.1%	4.1%	12.2%	
	IVB	*N*	0	0	0	0	1	1	
		%	0.0%	0.0%	0.0%	0.0%	2.0%	2.0%	
Surgical margins infiltration	Negative	*N*	16	13	15	6	7	57	**0.021**
		%	25.4%	20.6%	23.8%	9.5%	11.1%	90.5%	
	Positive	*N*	0	0	2	2	2	6	
		%	0.0%	0.0%	3.2%	3.2%	3.2%	9.5%	
Surgical parametric infiltration	Negative	*N*	16	12	16	6	8	58	0.056
		%	25.4%	19.0%	25.4%	9.5%	12.7%	92.1%	
	Positive	*N*	0	1	1	2	1	5	
		%	0.0%	1.6%	1.6%	3.2%	1.6%	7.9%	
Histotype	Squamous	*N*	13	12	13	6	12	56	0.373
		%	19.4%	17.9%	19.4%	9.0%	17.9%	83.6%	
	Adenocarcinoma	*N*	4	1	3	1	0	9	
		%	6.0%	1.5%	4.5%	1.5%	0.0%	13.4%	
	Neuroendocrine	*N*	0	0	1	1	0	2	
		%	0.0%	0.0%	1.5%	1.5%	0.0%	3.0%	
Grade	1	*N*	1	1	0	0	0	2	0.699
		%	1.5%	1.5%	0.0%	0.0%	0.0%	3.0%	
	2	*N*	7	2	5	4	6	24	
		%	10.4%	3.0%	7.5%	6.0%	9.0%	35.8%	
	3	*N*	10	10	12	4	5	41	
		%	14.9%	14.9%	17.9%	6.0%	7.5%	61.2%	
pN Status	0	*N*	18	11	11	0	0	40	** < 0.001**
		%	26.5%	16.2%	16.2%	0.0%	0.0%	58.8%	
	≥ 1	*N*	0	2	6	8	12	28	
		%	0.0%	2.9%	8.8%	11.8%	17.6%	41.2%	
Age (range)	59.67 (31–84)	58.77 (37–72)	51.59 (35–71)	62.50 (39–82)	51.17 (36–69)	56.31 (31–84)	0.107		
Tumor size (range)	39.40 (17–94)	43.15 (15–75)	48.29 (32–72)	57.13 (35–90)	57.75 (45–75)	47.41 (15–94)	**0.036**

**Table 3:** Node-RADS score performance in predicting pN status in cervical cancer

	Sensitivity (CI 95%)	Specificity (CI 95%)	PPV (CI 95%)	NPV (CI 95%)	Accuracy (CI 95%)
≥ 2	100.00% (87.66% to 100.00%)	45.00% (29.26% to 61.51%)	56.00% (49.02% to 62.75%)	100.00% (81.47% to 100.00%)	67.65% (55.21% to 78.49%)
≥ 3	92.86% (76.50% to 99.12%)	72.50% (56.11% to 85.40%)	70.27% (58.58% to 79.80%)	93.55% (79.00% to 98.24%)	80.88% (69.53% to 89.41%)
≥ 4	71.43% (51.33% to 86.78%)	100.00% (91.19% to 100.00%	100.00% (83.16% to 100.00%)	83.33% (73.57% to 89.98%)	88.24% (78.13% to 94.78%)
5	42.86% (24.46% to 62.82%)	100.00% (91.19% to 100.00%)	100.00% (73.54% to 100.00%)	71.43% (64.46% to 77.51%)	76.47% (64.62% to 85.91%)

The inter-observer agreements between the Node-RADS scores assigned by the senior reader compared with the scores assigned by junior reader 1 and the scores assigned by the senior reader compared with junior reader 2 were 0.888 and 0.738, respectively.

A total of 78 LNs with Node-RADS score ≥ 3 from 27 patients were considered for statistical analysis of ADC, cADC and rADC values: 54/78 were positive at definitive histology (69.2%) while 24/78 were negative at definitive histology (30.8%).

The Kruskal–Wallis test showed that ADC, cADC and ADC values were significantly different among Node-RADS 3, 4 and 5 patients (ADC: *p* = 0.01; cADC: *p* = 0.02; rADC: *p* = 0.036) (Table 4).

**Table 4:** ADC values in Nodes-RADS 3, 4 and 5 presented as mean (± standard deviation), median, minimum and maximum values

	NODE-RADS			
	3	4	5	Total
Mean ADC (× 10^−3^ mm^2^/s)				
Mean ± SD	0.968 ± 0.171	0.849 ± 0.131	0.807 ± 0.144	0.901 ± 0.170
Median	0.975	0.875	0.811	0.899
Range	0.603–1.458	0.430–1.066	0.470–1.043	0.430–1.458
cADC				
Mean ± SD	0.739 ± 0.163	0.707 ± 0.218	0.578 ± 0.118	0.693 ± 0.180
Median	0.73	0.646	0.594	0.6579
Range	0.43–1.14	0.5–1.48	0.32–0.83	0.32–1.48
rADC (× 10^−3^ mm^2^/s)				
Mean ± SD	0.50 ± 0.227	− 0.012 ± 0.124	− 0.140 ± 0.361	0.009 ± 0.255
Median	0.99	− 0.012	− 0.009	0.034
Range	− 0.645 to 0.465	− 0.281 to 0.175	− 1.066 to 0.19	− 1.066 to 0.465

**Fig. 6:**
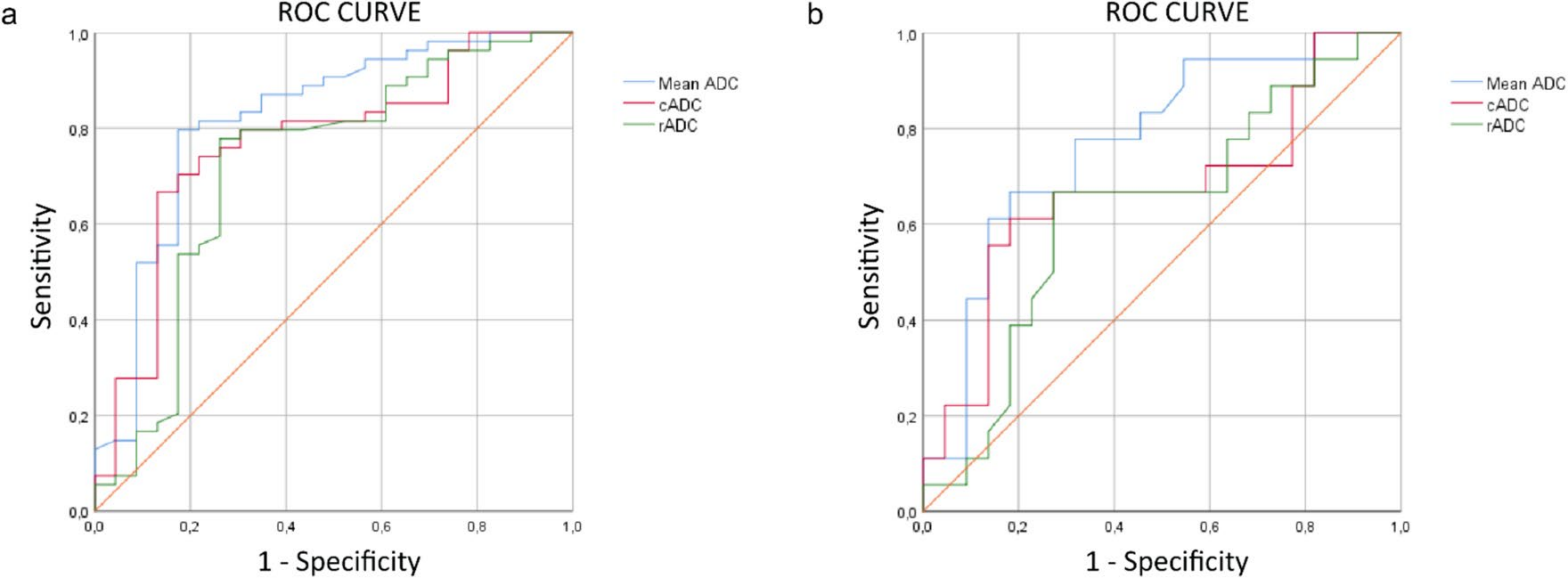
**a** Receiver operating characteristic (ROC) curve for the prediction of pN status using ADC values in lymph nodes with Node-RADS score ≥ 3. Area under the curve (AUC), mean ADC = 0.820; cADC = 0.772; rADC = 0.723. **b** ROC curve for the prediction of pN status using ADC values in lymph nodes with Node-RADS score = 3. Area under the curve (AUC), mean ADC = 0.771; cADC = 0.677; rADC = 0.631

The prediction of N status in Node-RADS ≥ 3 group corresponded to an AUC of 0.820 for ADC values, 0.772 for cADC values, and 0.723 for rADC values (Fig. 6). Based on the Youden index of AUCs of ADC-based values for Node-RADS ≥ 3, the following thresholds were used: ADC < 0.958 × 10^−3^ mm^2^/s; rADC < 0.087 × 10^−3^ mm^2^/s; cADC < 0.71. ADC values performances in predicting pN status in LNs with Node-RADS score ≥ 3 are reported in Table 5.

**Table 5:** ADC values performance in predicting pN status in cervical cancer in lymph nodes with Node-RADS ≥ 3

	Sensitivity (CI 95%)	Specificity (CI 95%)	PPV (CI 95%)	NPV (CI 95%)	Accuracy (CI 95%)	AUC	Threshold
Mean ADC (× 10^−3^ mm^2^/s)	81.48% (68.57% to 90.75%)	78.26% (56.30% to 92.54%)	89.80% (80.04% to 95.08%)	64.29% (49.71% to 76.63%)	80.52% (69.91% to 88.67%)	0.820	< 0.958
cADC	74.07% (60.35% to 85.04%)	73.91% (51.59% to 89.77%)	86.96% (76.70% to 93.10%)	54.84% (42.12% to 66.96%)	74.03% (62.77% to 83.36%)	0.772	< 0.087
rADC (× 10^−3^ mm^2^/s)	77.78% (64.40% to 87.96%)	69.57% (47.08% to 86.79%)	85.71% (76.09% to 91.88%)	57.14% (43.05% to 70.17%)	75.32% (64.18% to 84.44%)	0.723	< 0.71

AUC, area under the ROC curve

The prediction of N status in the Node-RADS 3 group corresponded to an AUC of 0.771 for ADC values, 0.677 for cADC values, and 0.631 for rADC values (Fig. 6). According to the Youden index of ADC AUCs for Node-RADS score 3, the following thresholds were used: ADC < 0.958 × 10^−3^ mm^2^/s; rADC < 0.073 × 10^−3^ mm^2^/s; cADC < 0.68. ADC values performances in predicting pN status in LNs with Node-RADS score 3 are reported in Table 6.

**Table 6:** ADC values performance in predicting pN status in cervical cancer in lymph nodes with Node-RADS = 3

	Sensitivity (CI 95%)	Specificity (CI 95%)	PPV (CI 95%)	NPV (CI 95%)	Accuracy (CI 95%)	AUC	Threshold
Mean ADC (× 10^−3^ mm^2^/s)	78.57% (59.05% to 91.70%)	81.82% (59.72% to 94.81%)	84.62% (68.94% to 93.16%)	75.00% (58.96% to 86.23%)	80.00% (66.28% to 89.97%)	0.771	< 0.958
cADC	61.11% (35.75% to 82.70%)	72.73% (49.78% to 89.27%)	64.71% (45.77% to 79.93%)	69.57% (54.82% to 81.15%)	67.50% (50.87% to 81.43%)	0.677	< 0.073
rADC (× 10^−3^ mm^2^/s)	66.67% (40.99% to 86.66%)	68.18% (45.13% to 86.14%)	63.16% (46.15% to 77.42%)	71.43% (55.07% to 83.61%)	67.50% (50.87% to 81.43%)	0.631	< 0.68

AUC, area under the ROC curve

## Discussion

Prior to 2018, CC staging followed the clinical FIGO system, which did not incorporate LN status, despite its significant impact on patient prognosis and management [9].

In 2018, the FIGO classification underwent revision to include imaging and pathological findings, leading to the inclusion of LN status assessment and the introduction of stage IIIC [10–12].

Subsequently, in 2019, the European Society of Urogenital Radiology (ESUR) established a working group to update imaging guidelines in alignment with the 2018 FIGO system [3]. The revised ESUR guidelines emphasize the pivotal role of MRI, particularly T2-weighted imaging and DWI-MR for staging, monitoring treatment response, and evaluating disease recurrence [13, 14].

LN involvement has thus become critical in CC staging, representing a crucial prognostic indicator and influencing the decision between conservative chemoradiation and surgical resection.

Consequently, enhancing diagnostic accuracy in detecting metastatic LNs is mandatory to establish prognosis and determine the most suitable treatment strategies, reducing patient morbidity.

Currently, histological examination following laparotomic lymphadenectomy remains the gold standard for defining LN metastasis in patients with CC. However, this invasive procedure is associated with some risks, including bleeding, infection, and lymphedema of the lower extremities. Thus, to enhance the accuracy of LN metastasis prediction and mitigate associated complications, there is a critical need for a non-invasive approach that achieves high levels of precision. Such an approach would not only enhance diagnostic efficacy but also facilitate informed decision making regarding surgery, ultimately reducing morbidity rates related to the procedure.

18-Fluorodeoxyglucose positron emission tomography/CT (FDG-PET/CT) currently stands as the most precise imaging modality for LN staging. A recent meta-analysis by Ruan et al. [15] revealed a sensitivity of 72% and a specificity of 96% for FDG-PET/CT. Although FDG-PET/CT plays a pivotal role in detecting LN metastases, it presents disadvantages such as high cost, significant radiation exposure, and limited spatial resolution, which limit its utility. Furthermore, the presence of micro-metastases may compromise the diagnostic accuracy of FDG-PET/CT, making histological examination irreplaceable [16].

Conversely, MRI offers a non-invasive alternative and may be more widely accessible in some clinical settings [11, 12]. Pathological LN detection with MRI primarily relies on dimensional criteria (≥ 10 mm on the short axis (SA)), exhibiting a sensitivity raging from of 56 to 61% and specificity of 89 to 91%. However, lowering the threshold to 8 mm may increase sensitivity at the expense of specificity. In addition to dimensional parameters, morphological characteristics such as heterogeneous signal intensity (SI), spiculated margins, and asymmetry relative to the contralateral side can enhance sensitivity [17, 18].

Recently, the Node-RADS classification system has been introduced with the aim of standardizing radiological terminology for identifying pathological LNs across all malignancies, utilizing both CT and MRI. In this study, the diagnostic performance of Node-RADS in identifying metastatic LN involvement in patients with CC was assessed by comparing assigned scores with the final histological examination. The accuracy of Node RADS in identifying malignant features and detecting positive LNs was therefore evaluated.

Our findings revealed a significant positive correlation between Node-RADS score and histological LN findings, affirming its precision. Additionally, a positive correlation was observed with preoperative and postoperative FIGO stage on MRI, as well as LN size, indicating that higher risk LNs corresponded to larger LNs and increased tumor infiltration. Similarly, a positive correlation was found between Node-RADS score and positive resection margins, likely due to the higher incidence of pathological LNs in locally advanced CC.

Node-RADS exhibited good sensitivity and high NPV in predicting the low risk of LN malignancy in Node-RADS ≥ 2 and Node-RADS ≥ 3 cut offs. Conversely, high specificity and NPP were observed at Node-RADS ≥ 4 and Node-RADS 5 cut-offs.

The Node-RADS ≥ 3 cut-off identified a subgroup of definitely pathological LNs (PPV = 100%), encompassing those classified as Node-RADS 4 and 5, who yielded maximal specificity (100%). However, this subgroup also included Node-RADS score 3 LNs, reducing overall specificity (72.5%) due to false positive LNs (PPV = 70.3%).

Thus, the best cut-off for distinguishing benign from pathological LNs is Node-RADS ≥ 3, facilitating discrimination between negative (Node-RADS 1–2) and positive LNs. While Node-RADS 4 and 5 groups exhibit high accuracy for pathological LNs, the risk of malignancy in the Node-RADS 3 group presents a challenge for patient categorization.

Indeed, consideration of treatment for patients with suspected metastatic disease to LNs remains crucial, as the number and location of LN involvement have a significant impact on prognosis, with 5-year disease-specific survival (DSS) rates for patients with none, one, and multiple LN metastases of 87%, 84%, and 61%, respectively [19, 20].

The present study aligns with findings from two prior studies examining the utility of the Node-RADS score in characterizing LNs among patients with prostate [6] and bladder cancer [7]. The first study reported high specificity but low sensitivity of Node-RADS in predicting LN involvement by malignancy. Conversely, the second study demonstrated moderate to high diagnostic accuracy in detecting LN invasion and proposed different cut-offs tailored to specific clinical contexts. Additionally, Node-RADS ≥ 3 emerged as the optimal cut-off in the bladder cancer study, with a negative predictive value of 90% along with high sensitivity and specificity at both patient (78.6% and 77.1%, respectively) and LN (82.4% and 93.9%, respectively) levels [7]. These findings are in line with the outcomes of the current investigation.

The Node-RADS classification relies on dimensional criteria and morphological features, lacking incorporation of DWI sequences, which may be a limitation.

In fact, multiple studies have underscored the significance of DWI-MRI as an important additional tool for identifying involved LNs, discriminating reactive phenomena, such as peritumoral edema from real neoplastic involvement, and facilitating assessment of the spread and size of the neoplasm [19, 20]. The high b-value of DWI-MR enhances LNs, rendering them prominently visible with high SI against a low SI background. Although metastatic LNs typically exhibit significantly lower ADC values compared to benign LNs, the use of variable cut-offs and the notable overlap of ADC values limit routine clinical applicability [21, 22].

However, the application of ADC values (ADC, rADC, cADC) has been demonstrated to enhance differentiation between metastatic and hyperplastic pelvic LNs in patients with CC, aiding in the characterization of pathological LNs (Node-RADS 3, 4 and 5).

Among the three calculated ADC values, mean ADC emerged as the most diagnostic parameter, with a sensitivity of 81.48%, specificity of 78.26%, PPV of 89.80% and NPV of 64.29% in predicting the status of LNs categorized as Node-RADS ≥ 3 (Table 5), with AUC values of 0.820 and 0.771 for Node-RADS ≥ 3 and Node-RADS 3, respectively (Tables 5 and 6).

Notably, ADC cut-off values of 0.958 × 10^−3^ mm^2^/s and 0.958 × 10^−3^ mm^2^/s were determined for Node-RADS ≥ 3 and Node-RADS 3, respectively.

In contrast to our study, in the literature some authors reported different findings.

Chen et al. [23] conducted a study measuring both ADC and rADC, using histology as the reference standard. Histological assessment was performed on tissue samples obtained from patients before and after neoadjuvant radiotherapy and/or chemotherapy. The authors identified ADC and rADC cut-off values of 1.15 × 10^−3^ mm^2^/s and 0.28 × 10^−3^ mm^2^/s, respectively, exhibiting comparable sensitivity and specificity to our results (ADC sensitivity = 83.3%, specificity = 74.7%; rADC sensitivity = 80.3%, specificity = 72.4%).

Kim et al. [24] analyzed 680 LNs from 143 patients who underwent hysterectomy and lymphadenectomy, without specifying the FIGO staging of the patients. They determined an ADC cut-off value of 0.911 × 10^−3^ mm^2^/s, demonstrating good sensitivity (83%) but lower than the one based on dimensional criteria using SA measurements (91%).

Gui et. al. [25] assessed the utility of ADC-based criteria (ADC, rADC, and cADC) in characterizing pelvic metastatic LNs in 34 patients with locally advanced CC, utilizing PET/CT as the reference standard. They observed that both mean ADC and rADC values of metastatic (PET/CT-positive) LNs were significantly lower than those of non-metastatic (PET-CT negative) LNs.

Our study has some limitations. While DWI and ADC at the pelvic level are recognized for their important role in assessing metastatic LNs, several LNs were excluded from the final analysis due to challenges in calculating ADC parameters. The primary difficulty came from the analysis of bulky LNs with central necrosis, where liquefactive necrosis allows unrestricted water molecule movement. Additionally, measurement difficulties arose from technical limitations or motion artifacts. Furthermore, the inclusion of patients who underwent NACT prior to surgery may have led to regression of LN metastases on histological examination, resulting in potential discordance between the risk determined by preoperative MRI examination with the Node-RADS score and the final complete histological LNs assessment. Moreover, this is a retrospective study with a relatively small sample size, with heterogeneous histological types of CC.

On the other hand, inter-reader agreement ranged from good to excellent, even among less experienced radiologists in gynecological imaging. Consequently, the high agreement among readers suggests the applicability of the Node-RADS scoring system across different degrees of experience in pelvic MRI, enhancing its utility in the clinical practice and structured reporting, even for novice radiologists.

Our study focused exclusively on MRI images, yet Node-RADS can be potentially applied to CT imaging as well. Further studies should examine whether the diagnostic performance of Node-RADS differs between these imaging modalities.

Moreover, integration of the Node-RADS system with other clinical features, such as patient age, tumor size, presence of lymphovascular space invasion, histological tumor type, degree of cell differentiation, and parameter involvement, could enhance LN involvement detection. Furthermore, inclusion of additional imaging parameters such as long axis size, long to short axis ratio, and LN volume could improve the diagnostic performance in LN characterization and be incorporated into the Node-RADS score. The ultimate goal would be to develop predictive models that consider all these factors individually to optimize treatment planning tailored to each patient.

## Conclusion

MRI plays a key role in the evaluation of CC, exhibiting high diagnostic accuracy in assessing tumor extent and LN involvement. Our study shows the efficacy of the Node-RADS score as a reliable system for standardized evaluation of LN stations, improving the classification of the N-parameter.

DWI-RM and assessment of the ADC parameter have emerged as useful tools in characterizing the N parameter and consequently detecting LN metastasis. In agreement with literature data, our study emphasizes the importance of the quantitative information provided by ADC in differentiating between non-specific and pathological LNs. Specifically, mean ADC emerged as the optimal diagnostic parameter for differential diagnosis, in comparison with relative and corrected ADC, particularly in “intermediate-risk” and “high-risk” LNs categorized in the Node-RADS ≥ 3 group.

Nonetheless, large prospective multicenter studies are needed to validate the results obtained from our investigation.

## Abbreviations

CC: Cervical cancer
RADS: Reporting and data system
LN: Lymph node
MRI: Magnetic resonance imaging
DWI: Diffusion-weighted imaging
ADC: Apparent diffusion coefficient
FOV: Field of view
NACT: Neoadjuvant chemotherapy
FIGO: International Federation of Gynecology and Obstetrics
CT: Computed tomography
SA: Short axis
SI: Signal intensity
